# A tunable electron beam source using trapping of electrons in a density down-ramp in laser wakefield acceleration

**DOI:** 10.1038/s41598-017-12560-8

**Published:** 2017-09-25

**Authors:** Henrik Ekerfelt, Martin Hansson, Isabel Gallardo González, Xavier Davoine, Olle Lundh

**Affiliations:** 1 Department of Physics, Lund University, P.O. Box 118, S-22100 Lund, Sweden; 2 CEA, DAM, DIF, Bruyères-le-Châtel, 91297 Arpajon, France

## Abstract

One challenge in the development of laser wakefield accelerators is to demonstrate sufficient control and reproducibility of the parameters of the generated bunches of accelerated electrons. Here we report on a numerical study, where we demonstrate that trapping using density down-ramps allows for tuning of several electron bunch parameters by varying the properties of the density down-ramp. We show that the electron bunch length is determined by the difference in density before and after the ramp. Furthermore, the transverse emittance of the bunch is controlled by the steepness of the ramp. Finally, the amount of trapped charge depends both on the density difference and on the steepness of the ramp. We emphasize that both parameters of the density ramp are feasible to vary experimentally. We therefore conclude that this tunable electron accelerator makes it suitable for a wide range of applications, from those requiring short pulse length and low emittance, such as the free-electron lasers, to those requiring high-charge, large-emittance bunches to maximize betatron X-ray generation.

## Introduction

In 1979^1^, Tajima and Dawson proposed using a plasma as an accelerating structure for electrons. A focused laser pulse can excite a plasma wave due to the fact that the high intensity laser pulse acts as an electron plough, creating an ion cavity in its wake. This cavity is quickly replenished with electrons which results in a plasma wave trailing the laser pulse. The phase velocity of the plasma wave is close to the group velocity of the laser pulse. The plasma wave transforms the transverse electric fields of the laser into strong longitudinal electric fields that can reach hundreds of GV/m. These fields can in turn be used to accelerate charged particles. This concept is now referred to as laser wakefield acceleration (LWFA). In 2004, the first quasi-monoenergetic beams were produced using this technique without an external electron injection source^2–4^. When the driving laser pulse is strong enough, the ion cavity is commonly referred to as a bubble^5^, which is the terminology that will be used to refer to the plasma wave behind the laser pulse in this report.

A density down-ramp is a region where the background density of the plasma is making a transition from a higher to a lower electron density in the direction of laser pulse propagation. When a laser pulse travels through a density down-ramp, the trailing bubble will elongate, since the size of the bubble scales as ${n}_{e}^{-\mathrm{1/2}}$, where *n*_*e*_ is the background electron number density^6^. The elongation can, under certain conditions, trigger wave breaking at the back of the bubble; that in turn causes transverse electron injection. This scheme was first proposed by Bulanov *et al*.^7^, who considered a slow density transition, and later by Suk *et al*.^8^ who suggested using a sharper transition. It has since then been studied both experimentally and numerically^9–17^. The effects of changing the down-ramp length (by varying the gradient) have been studied earlier in detail by Samant *et al*.^18^. They propose to use the density down-ramp injection as an injector for a soft X-ray free-electron laser (FEL). In a recent paper, Massimo *et al*.^19^ conducts a numerical study on a more shock-like profile where they also vary the peak density. Both papers find results that are in agreement with ours. In this paper, we further explain the physical processes and provide scaling laws for some of the electron bunch properties.

In previous experimental studies^10,13^, it was shown that density down-ramp injection is ideal for reproducibility. It allows for control of the electron bunch charge, by tuning the density difference between the two density regions, and the bunch energy, by controlling the length of the plasma after the density down-ramp.

Today, LWFA is one of the most promising compact accelerator techniques. There are many different proposed applications of the electron beams and/or betatron X-ray beams produced during the acceleration. Each application has its preferred set of electron bunch parameters. For example, to use the produced electron bunch as an injector for a compact FEL, one would typically prefer a short electron bunch with low emittance, low energy spread and high current^20^. However, if one would like to utilize the betatron (synchrotron-like) X-rays produced during the acceleration process, other characteristics such as high charge and large emittance (larger betatron oscillations) are preferred^21^.

In this report, we present a numerical study on the effect of down-ramp injection on the electron bunch parameters. The focus of the report lies on investigating the injection process and therefore the setup is kept constant until the density down-ramp. It is shown that the amount of charge in the electron bunch can be tuned by two parameters in the density profile; the density difference between two density regions where injection occurs as well as the steepness of the density down-ramp. The mean electron bunch energy and energy spread for different cases are presented and compared at a given distance. Furthermore, the trapped electrons fill up the bubble from the back along the optical axis as it expands. This allows for control of the electron bunch duration/length. Lastly, it is shown that the divergence of the trapped electron bunch depends on the steepness of the density down-ramp.

This study was performed using the quasi-cylindrical particle-in-cell code CALDER-Circ^22^. This code decreases the computational load by exploiting the cylindrical symmetry of the LWFA process. The numerical parameters for the simulations are given in the Method section.

## Results

**Figure 1:**
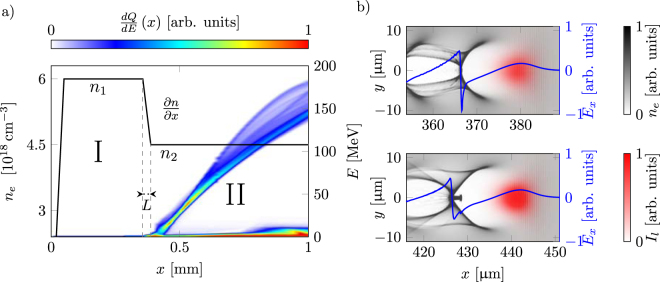
(**a**) A typical density profile used in the simulations. The profile is divided into a high - density region (I), characterized by the density *n*_1_, and a low - density region (II), characterized by the density *n*_2_. They are connected through a density down-ramp characterized by the gradient ∂*n*/∂*x* and its length *L*. The evolution of the energy spectrum *dQ*/*dE*(*x*) of the electron bunch as a function of the longitudinal position *x* of the laser pulse’s peak is shown as a density map in the background, where *Q* is the beam charge and *E* the beam energy. It can be seen that the electron bunch is trapped at the density down-ramp and then accelerated throughout the lower plasma density region. (**b**) Two snapshots from a typical simulation. A plasma wave has been excited to the bubble-regime by the laser pulse. The background electron density *n*_*e*_ is shown in grayscale, the laser intensity *I*_*l*_ is shown by the red color scale, and the longitudinal electric field *E*_*x*_ on the optical axis is shown as the blue curve in the graph. The upper plot is a snapshot from just before the laser pulse enters the density down-ramp. The lower plot shows a snapshot after the laser pulse has passed the down-ramp and traveled into the second region of constant density. The bubble expands in the density down-ramp and as a consequence, electrons are trapped along the optical axis from the back of the bubble, and begin to accelerate.

The density profile of a typical simulation is illustrated in Fig. 1a. The plasma profile is composed of an 18-μm long entrance ramp to the first plateau density (region I) characterized by the density *n*_1_ which extends for 340 μm, followed by a density down-ramp to the second plateau (region II) characterized by the density *n*_2_ which ends after 840 μm. The down-ramp between region I and II is characterized by its length *L* and its gradient ∂*n*/∂*x* = (*n*_1_ − *n*_2_)/*L*. In this study we vary *L*, *n*_2_, and consequently ∂*n*/∂*x*. All other parameters are kept constant in order to as well as possible differentiate all the different mechanisms at play. This ensures that an identical laser pulse enters the down-ramp in each simulation. The plasma profile is 130 μm wide in both transverse directions with no density gradients. *n*_1_ is kept constant to keep the laser parameters fixed when the laser pulse enters the down-ramp. We emphasize that alternatively, *n*_1_ could be varied to achieve similar results for the charge, bunch length, and emittance of the electron bunch. However, this would also change the laser pulse’s evolution, and it would be hard to isolate what effects comes from changing the down-ramp parameters. Varying *n*_2_ greatly impacts the acceleration process since the accelerating fields change.

A typical simulation is illustrated with snapshots from one of the simulations in Fig. 1b. Note that the laser pulse effectively excites a bubble by the end of region I yet no injection has occurred until this point. The electrons are injected at the back of the first bubble during the density down-ramp by so-called transverse-injection. When the back of the bubble has reached the end of the down-ramp the injection stops. In region II, the laser pulse still effectively drives a bubble with injected charge.

For the simulations the following laser parameters were used: a normalized vector potential *a*_0_ = 1.8, a Gaussian laser focus with a full width at half maximum (FWHM) width of 18 μm, and a temporal Gaussian with *t*_*FWHM*_ = 30 fs. The focal plane of the laser beam in vacuum was located at the beginning of region I, 20 μm into the plasma.

In this section we present results that will show that modifying the properties of the density down-ramp is a promising method for controlling the electron bunch’s charge, length and transverse emittance. The results presented in this report are only valid if we are in or close to the bubble regime: the laser pulse length and waist are in the same range, *a*_0_ is higher than 2, the plasma densities are chosen so that the laser pulse length is smaller than the plasma wavelength. The densities should also be high enough to ensure self-focusing and guidance of the laser pulse over several Rayleigh lengths.

### Amount of accelerated charge and bunch energy

**Figure 2:**
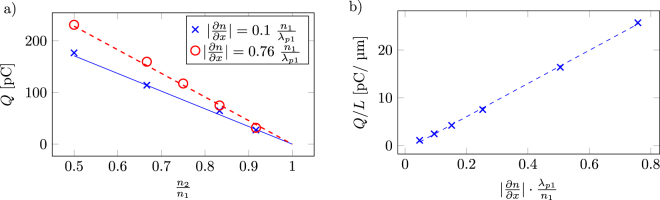
(**a**) Trapped charge as a function of the lower density value for two different gradient slopes. The symbols show data taken from the simulations. The corresponding lines are Eq. (2) with *k*_1_ = 34.761*λ*_*p*1_/(*n*_1_μm) pC and *k*_2_ = −0.912 pC/μm. (**b**) Trapped charge over down-ramp length as a function of different density down-ramp gradients with *n*_2_/*n*_1_ = 0.5. The amount of charge per length increases with the steepness of the gradient. The dashed blue line is Eq. (2) plotted with *k*_1_ = 34.76*λ*_*p*1_/*(﻿n*_1_μm) pC and *k*_2_ = −0.91 pC/μm.

As can be seen in Fig. 1a, a quasimonoenergetic beam without a low-energy tail is accelerated to more than 100 MeV after 0.5 mm propagation in region II. The electron bunch parameters are measured 0.9 mm into the plasma for different down-ramp lengths and density differences. The amount of trapped charge was measured for down-ramps with different density slopes ∂*n*/∂*x* and density ratios *n*_2_/*n*_1_. The results are plotted in Fig. 2a.

As is clear from Fig. 2a, the amount of charge have a quasi-linear dependence on the density difference. The trend shows that the amount of charge injected in a density down-ramp where the density difference is varied can be described by a very simple linear relation for the beam charge *Q*:

1 $$Q=k(\frac{\partial n}{\partial x})({n}_{1}-{n}_{2})=k(\frac{\partial n}{\partial x})\frac{\partial n}{\partial x}L$$

where *k*(∂*n*/∂*x*) is a function of the density slope and the laser properties. This relation agrees with previous experimental findings^13^. Since *n*_1_ is close to the self-injection threshold, injection is reached even for small density differences, in contrast to the cases studied by Massimo *et al*.^19^.

In Fig. 2b, the average amount of injected charge per μm (the total injected charge *Q* over the down-ramp length *L*) is plotted for different density slopes with a fixed density ratio of *n*_2_/*n*_1_ = 0.5. As can be seen, the beam charge over the down-ramp length scales nearly linearly with the slope. However, the higher charge per length unit with steeper gradient is compensated by the fact that injection occurs over a shorter distance, as an increase in steepness by some factor also decrease the gradient length L by the same factor for the same density difference. Note that, in Fig. 2a, as the slope steepness ∂*n*/∂*x* is increased almost one order of magnitude, the amount of charge increases by 25% in total for the density ratio of *n*_2_/*n*_1_ = 0.5. Using a linear fit, *Q*/*L* = *k*_1_(∂*n*)/(∂*x*) + *k*_2_, from Fig. 2b, one can describe the charge dependence for the different slopes by making it a function of the down-ramp length *L* and adding the linear correction term *k*_2_

2 $$Q={k}_{1}\frac{\partial n}{\partial x}L+{k}_{2}L\mathrm{.}$$

Here, *k*_1_ is not dependent on the gradient slope and *k*_2_ is negative. For our specific laser and plasma conditions, we have found that *k*_1_ = 34.76 *λ*_*p*1_/(*n*_1_ μm) pC and *k*_2_ = −0.91 pC/μm. To test the validity of the model, Eq. (2) (with different *k*_1_ and *k*_2_) was tested on the data presented by Samant *et al*.^18^ and Massimo *et al*.^19^. It is found that the relation can be used to describe the trend of injected charge for the cases where the down-ramp steepness ∂*n*/∂*x* > = 2 ⋅ 10^16^ cm^−3^ μm^−1^.

The setup of this numerical experiment, keeping *n*_1_ fixed and varying the acceleration density *n*_2_ impacts the acceleration process. The acceleration part is intimately linked to the injection, through the chosen value of *n*_2_, which will directly affect the laser evolution, the accelerating field and the dephasing length. It will also have impact on the beamloading effects that depends on *n*_2_ and the beam charge/current. Even though this paper is mainly focused on the beam parameters that directly results from the injection part of the process, in particular the electron bunch length, charge, and transverse emittance; for completeness, we also provide information on the electron bunch energy and energy spread for the different scenarios.

**Figure 3:**
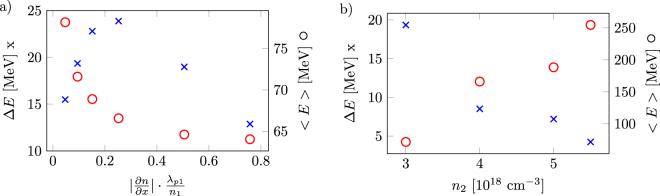
(**a**) In blue, energy-spread (root mean square error from mean energy) as a function of the different down-ramp steepness. In red the mean energy is plotted for a fixed lower density of *n*_2_ = 3 ⋅ 10^18^ cm^−3^. (**b**) In blue, energy spread for different lower densities *n*_2_. In red mean energy for the same simulations with a fixed steepness of 0.1 (*n*_1_)/(*λ*_*p*1_). These values are measured 0.9 mm into the plasma.

In Fig. 3a the energy spread and mean energy for different gradient slopes with *n*_2_ = 3 ⋅ 10^18^ cm^−3^ is presented. The energy spread is lowest for the shortest down-ramp. The trapping of the electrons in the steepest gradient takes place over the shortest amount of time, thus the electrons are accelerated for almost the same amount of time. The mean energy increases with a decrease in the gradient slope due to the fact that the average background density including the density down-ramp is higher, and more charge in the steeper cases decreases the accelerating field due to a stronger beamloading effect.

In Fig. 3b the energy spread and mean energy for different gradient lengths with (∂*n*)/(∂*x*) = 0.1 (*n*_1_)/(*λ*_*p*1_) is shown. Note that caution should be used before interpreting this comparison since the values are measured at the same position, and not at the dephasing length for each acceleration process.

### Control of electron bunch length

In density down-ramp injection, the electron bunch length is expected to be equal to the difference between the bubble lengths in the high and low density regions given that the injection occurs throughout the down-ramp. It is therefore important to measure and estimate this bubble length and growth as precisely as possible. From Fig. 1b it is clear that the pulse length of the trapped electrons is the same as the elongation of the wakefield, indicating the possibility of predicting the length of the electron bunch. Similar effects can be seen in Massimo *et al*.^19^ in which the effects of a varied peak density on the bunch length is presented. An analytical and numerical scaling law for the bubble radius *r*_*b*_ is given in a previous paper by Lu *et al*.^6^. In this paper^6^, the relation *r*_*b*_ = ${c}_{r}\sqrt{{a}_{0}}{\lambda }_{p}$ is derived. Here, *c*_*r*_ is a constant, *λ*_*p*_ = 2*πv*/*ω*_*p*_ where *v* ≈ *c* is the group velocity of the laser pulse and ${\omega }_{p}=\sqrt{\frac{{n}_{e}{q}_{e}^{2}}{{m}_{e}{\varepsilon }_{0}}}$ is the non-relativistic angular plasma - frequency. *n*_*e*_ is the electron density, *q*_*e*_ is the electron charge, *m*_*e*_ is the rest mass of the electron and *ε*_0_ is the vacuum permittivity.

The constant of proportionality *c*_*r*_ is determined from PIC-simulations for a special case when the laser beam duration, waist, amplitude, and the plasma density are all matched. These matched properties that are discussed in the same paper^6^ are hereafter referred to as a matched condition. However, in experimental conditions, a matched laser pulse can be hard to achieve for different reasons, such as limited available beam power or energy in comparison to the laser pulse duration. Furthermore, a laser pulse going through a density down-ramp can not be matched in both density regions.

The size of the bubble does not only depend on the local plasma density, but also on the amount of trapped charge. The simulations conducted for this study show that it is possible to predict the full width pulse length *L*_*eb*_ of the electron bunch with the relation

3 $${L}_{eb}={C}_{1}{\rm{\Delta }}{\lambda }_{p}+{C}_{2}{Q}^{2}\mathrm{.}$$

Here, Δ*λ*_*p*_ is the difference in *λ*_*p*_ for the two density regions, *C*_1_ is a unitless constant coupling the theoretical linear non-relativistic plasma wavelength *λ*_*p*_ to the true plasma wavelength, and *C*_2_ is a constant with units of length/charge ^2^. The second term *C*_2_ *Q* ^2^ is chosen to correct for the elongation of the bubble due to the beamloading effects observed in the simulation. *C*_1_ includes the dependence of *a*_0_ and is taken from the simulations with beamloading turned off, i.e., *C*_1_Δ*λ*_*p*_ is the true plasma wavelength difference between the two density regions without injected charge. How beamloading is turned off in the simulations is explained in the Method section.

**Figure 4:**
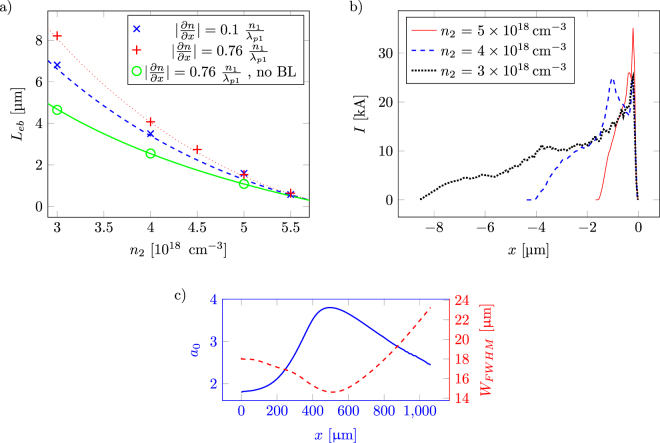
(**a**) The green circles correspond to the electron bunch length measurements with beamloading turned off. The green line is a fit using Eq. (3) with *C*_1_ = 0.83 and *C*_2_ = 0. The red plus signs are data points from the simulation of the electron bunch length, the dashed red curve represents a fit according to Eq. (3). The blue crosses and dotted line represents the same values for a gentler gradient slope. (**b**) Spatial electron density profiles of three different acceleration densities: *n*_2_ = 3, 4 and 5 ⋅ 10^18^ cm^−3^. Here, ∂*n*/∂*x* = 0.76*n*_1_/*λ*_*p*1_ with beamloading. (**c**) The peak normalized vector potential *a*_0_ (in blue) and FWHM width (dashed red) as function of the laser pulse’s peak position during a simulation with *n*_2_ = 3 ⋅ 10^18^ cm^−3^ and ∂*n*/∂*x* = 0.76*n*_1_/*λ*_*p*1_. For the different simulation, the peak value of *a*_0_ varies between 3.8 and 4.3, but the behaviour is the same for all simulations,* a*_0_ increases and the width of the pulse decreases.

The relation presented in Eq. (3) is displayed in Fig. 4a together with the electron beam lengths measured in the different simulations. The linear fits displayed in Fig. 2a that model the trapped charge are used to draw the predictive lines for the red and blue data sets. For both fits, *C*_1_ = 0.83 and *C*_2_ = 0.63 ⋅ 10^−4^ pC^−2^. *C*_2_ is calculated by fitting the difference in electron bunch length between the simulation with and without beamloading to the amount of charge squared. The quadratic dependence on *Q* was chosen because it allows for accurately fitting both curves with the same constant *C*_2_ while still yielding a simple predictive relation. From existing literature by Tzoufras *et al*.^23^ one would expect a linear dependence on Q, however, a quadratic dependence fits better with the simulation parameters in this report. Observe that the parameters in this report differentiates from the paper^23^ by Tzoufras *et al*. in two significant ways: The analysis by Tzoufras *et al*. is conducted for the matched regime and are making sure the beamloading is optimal. In this report, charge is injected while the background density (and thus the wakefield) changes while they are adding charge to a bubble in a constant background density. The green data set is taken from simulations where the beamloading effect is turned off, as discussed earlier. These simulations without beamloading effects almost perfectly follow the relation in Eq. (3) if *Q* = 0 and *C*_1_ = 0.83. The data points are taken for ∂*n*/∂*x* = 0.1 ${n}_{1}{\lambda }_{p1}^{-1}$ but there is no difference in bunch length for different ∂*n*/∂*x* when beamloading is turned off. Do note that the bunch length is significantly elongated by the beamloading effect on the wakefield, as can be seen in Fig. 4a.

In the theory from Lu *et al*. presented above, the bubble radius *r*_*b*_ scales with ${a}_{0}^{-\mathrm{1/2}}$. The variation of *a*_0_ due to laser self-focusing or laser beam evolution should thus play a role in the length of the electron bunch. The evolution of *a*_0_ and the FWHM beam waist of the laser is shown in Fig. 4c. However, as previously mentioned, this relation only holds for laser wakefield accelerators in a matched regime. In the matched regime, where the equation 2*πW*_0_/*λ*_*p*_ = *a*_0_ (*W*_0_ is the *e* ^−2^ laser beam waist) is fulfilled, *a*_0_ can not be changed without changing either *λ*_*p*_ or *W*_0_ accordingly. In the case presented in this report, *a*_0_ evolves due to a temporal and spatial compression of the laser pulse (as can be seen in Fig. 4c). The ratio *W*_0_/(*λ*_*p*_ *a*_0_) is therefore no longer constant, and the scaling laws of the matched regime are not applicable. The ponderomotive push on the electrons from the laser scales with *a*_0_ and an increase in bubble length can be expected from this effect. However, at the same time, the laser waist decreases and the laser pulse interacts with a smaller number of electrons. Thus, the widths of the plasma column that is pushed away decreases, which reduces the bubble length. It is important to note these effects counteract each other but the sum of these effects are not generally zero. Thus the model for the bubble radius, which assumes that ${r}_{b}\propto {a}_{0}^{\mathrm{1/2}}$, is not valid for this setup. To support this argument, a comparison between the bubble length before and after the down-ramp has been performed for different down-ramp lengths. The simulations show that for the cases with beamloading suppressed, there is no significant difference in the bubble length after the short and long gradients. At the same time, ${a}_{0}^{1/2}$ has increased by more than 10% for the longer gradient (*L* = 72 μm) due to the self-focusing effects from crossing a longer plasma region.

The longitudinal profile of three different electron bunches are shown in Fig. 4b. The charge distribution can be approximated as triangles, starting at approximately the same point in *x* − *dQ*/*dx* space and then a straight line can be drawn from this point to the end of the bunch length. This may be useful, as a triangular beam shape is required for optimal beamloading^23^, and consequently preservation of energy spread. However, for the parameters used in these simulations, optimal beamloading was not obtained.

### Controlling emittance

**Figure 5:**
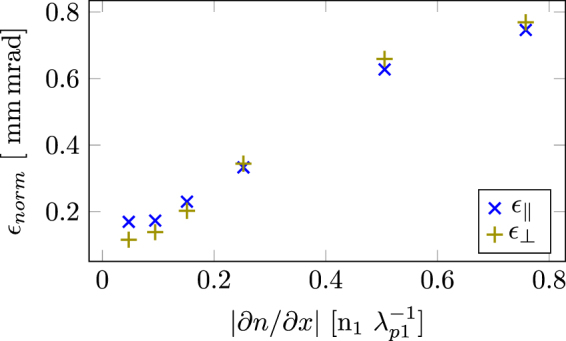
The normalized emittance as a function of the different density gradients. The normalized emittance scales with the sharpness of the density down-ramp. All the shown data is from simulations where *n*_2_ = 3 ⋅ 10^18^ cm^−3^. Here ${\varepsilon }_{\parallel }$ and *ε*_⊥_ is the normalized transverse emittance parallel and perpendicular to the polarization of the laser pulse.

From the simulations, it is clear that the divergence of the electron bunch also depends on the density gradient. The normalized emittance^24^ of electron bunches for different density gradients are shown in Fig. 5. A straight-forward relation can be seen between the steepness of the density gradient and the emittance. For a steep gradient, the back of the bubble slows down more rapidly, lowering the threshold for trapping, thus causing electrons with larger transversal momentum to be trapped.

**Figure 6:**
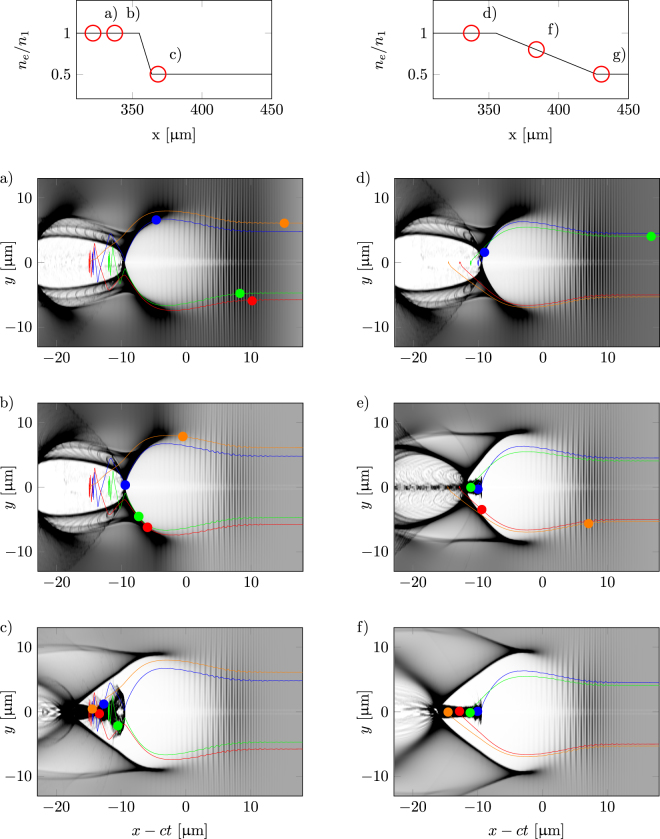
To the left, a series of 3 snapshots from the injection process for a steep gradient where ∂*n*/∂*x* = 0.76 ${n}_{1}{\lambda }_{p1}^{-1}$ with *L* = 9 μm. To the right, 3 snapshots from the injections process for a gentler gradient where ∂*n*/∂*x* = 0.1 ${n}_{1}{\lambda }_{p1}^{-1}$ with *L* = 72 μm. For both simulations *n*_2_ = 3 ⋅ 10^18^ cm^−3^. The trajectories are for 4 tracked macro particles from each process. The dot represent each macro particle's position in the frame at the time of the snapshot. As can be seen, for the steep gradient, injection is a complex process. Macro particles located in a defocusing and decelerating region are suddenly placed within the bubble structure as the bubble expands, and they can then be trapped. For the gentler gradient, the macro particles enters from the rear of the bubble as a continuous process throughout the gradient.

In Fig. 6, two sequences of density down-ramp injection are portrayed in the form of density sections from the simulations as the laser pulse passes through the down-ramp. The sequences are divided into the left and right side of the figure. They are from two different down-ramps: ∂*n*/∂*x* = 0.76 ${n}_{1}{\lambda }_{p1}^{-1}$ with *L* = 9 μm and ∂*n*/∂*x* = 0.1 ${n}_{1}{\lambda }_{p1}^{-1}$ with *L* = 72 μm, respectively. For both simulations *n*_2_ = 3 ⋅ 10^18^ cm^−3^. Every part of the sequence is accompanied by a number of tracked macro particles with their trajectories throughout the whole simulation plotted as lines. The dot on each line mark the position of the macro particle at the time of the depicted frame. From these examples, the difference in emittance is visually obvious.

It is clear that these simulations span over different injection regimes. The steep gradient (to the left) results in an electron beam with large oscillations from a non-trivial process. Macro particles injected almost at the same time are spread out over the electron bunch. The bubble expands rapidly over the plasma wave structure, and electrons are suddenly placed within the accelerating structure. Some of the trapped particles first enters in a decelerating and defocusing region behind the bubble, providing them with a big transverse momentum. However, due to the fast bubble expansion, these particles eventually reenter through the back of the bubble and become trapped, but keep a significant amount of their transverse momentum, increasing the overall beam transverse emittance. The gentler gradient (to the right in Fig. 6) gives rise to a smoother trapping of the macro particles over the course of the gradient where they align nicely after each other. The macro particles that are injected first are at the front of the bunch, the last to be injected are placed at the end of the bunch, in contrast to the steep gradient.

## Discussion

Described here is a numerical study on density down-ramps of different steepness and heights. Using only those two parameters, it is possible to tune three important parameters of the electron bunch. Given that the laser pulse is strong enough to drive a wakefield in both density regions, we should see similar effects for different densities and different density regions. It is also possible to vary the density *n*_1_. Care should be taken to keep the accelerating density *n*_2_ large enough to sustain a bubble and maintain a decent acceleration. In these simulations, it was clear that for densities below 3 ⋅ 10^18^ cm^−3^ the electron bunch was driving its own significant wakefield, ruining the acceleration process.

First, we have shown numerically that the electron beam charge depends quasi- linearly on the density jump Δ*n* in our case. However, a general expression for the injection cross section is hard to derive due to the complex nature of the wavebreaking process. As is demonstrated in this report, the injection process itself is affecting the expansion of the bubble through the beamloading effect. This makes the process rather complicated. Kostyokov *et al*.^25^ derives a cross section *σ* for self-injection in a matched regime

4 $$\frac{\sigma }{\pi }\approx 8({R}^{2}-{\frac{dR}{dt}}^{-1}),$$

where *R* is the bubble radius normalized to *c*/*ω*_*p*_. Comparing the results in this report to the model, we can not find a quantitative match. However, as the density down-ramp is getting steeper and *dR*/*dt* increases, the cross section *σ* increases which agrees well with the findings here.

To maximize the amount of charge in the electron bunch, a sharp density transition should be chosen. This comes at the price of a large emittance; however, if one wants a betatron source, this is ideal. From the particle trajectories in Fig. 6 it can be seen that that the trapping mecanism is different in the two different cases. In the gentler gradient, particles travelling along the edge of the bubble are trapped. This is well described by Kalmykov *et al*.^26^ where they present a model that states that the particles with the longest slippage time are the particles that are trapped. The slippage time is defined as the time that a particle interacts with the bubble. In other words, the particles that travels along the edge of the bubble until the back are the particles that get trapped first. The same paper also explains that as the change in density increases, the expansion rate of the bubble increases, and particles with shorter slippage time can be trapped. This implies that even particles that are experiencing decelerating and defocusing fields are trapped, and as can be seen from the trajectories, some of them enter the bubble with a relatively large radial momentum, which causes a large oscillation for the particle. The larger the emittance, the more oscillations, which produces more X-rays^21^.

The bunch length is an important quality of the electron bunch. For example, the produced X-ray pulse in a betatron source will not be shorter than the generating electron bunch^21^. It also affects the acceleration process in LWFA. In the blowout regime, the accelerating electric field is changing linearly with respect to the position in the bubble. This will cause an increase in energy spread for any extended electron bunch. The difference in energy gain will thus increase linearly with the electron bunch length. This might pose a problem for long electron bunches over an extended acceleration region as the energy spread will increase. Since the acceleration process starts earlier for the head of the electron bunch, those electrons will have higher energies in the beginning. Unless optimal beamloading^23^ is achieved, the tail of the electron bunch will gain more energy than the head due to phase-space rotation of the bunch. At some point during the acceleration process, the tail of the electron bunch will have gained roughly the same amount of energy that as the front part. If this happens, the bunch has reached a minimum in energy spread. For the simulation summarized in Fig. 1a, this happens when the average energy is around 40 MeV.

In conclusion, we show a possibility to tailor the parameters of the electron bunch produced in LWFA by tuning the density down-ramp. We demonstrate that the amount of charge, the bunch length, and the emittance can be controlled in a simple manner. However, these parameters are not fully decoupled and can therefore not be tuned independently.

## Methods

The results here are presented for a laser with a central wavelength of 800 nm. The density down-ramp is characterized by three parameters: *n*_1_ is defined as the electron density of the high-density plateau, *n*_2_ is the electron density of the low-density plateau where electrons are accelerated, and |∂*n*/∂*x*| is used to characterize the steepness of the gradient of the density down-ramp. The longitudinal plasma gradient is normalized to the upper plasma density and the theoretical linear non-relativistic plasma wavelength at that density. The parameters used to characterize the longitudinal density profile of the simulation is shown in Fig. 1a along with the longitudinal energy distribution of the accelerated electron and the density profile. In order to isolate the effects of the density down-ramp, the high density part *n*_1_ of the simulation was kept at a constant electron density of 6 ⋅ 10^18^ cm^−3^ throughout all simulations discussed in this paper.

The moving simulation box was kept large enough to simulate at least the first full plasma wave (or “bubble”) after the laser pulse in the low density region. The peak intensity of the laser pulse was kept at a distance of 23 μm behind the front boundary of the moving window. The cell-size of the simulation grid was set to be 16 nm in the longitudinal direction and 190 nm in the radial direction, with 3200 and 400 cells in the respective dimension. Each cell contained 48 macro particles. The integration timestep was set to 52 as. A third order spatial interpolation scheme was used. The macro particles were not randomly distributed but placed in a regular pattern evenly distributed in *x*, *r*, and *θ*. An anti-numerical Cherenkov Maxwell solver was used^27^. The fields are decomposed into three Fourier modes in the azimuthal direction. Test-particle diagnostics were used to determine the emittance of the accelerated electron bunch.

To be able to independently study the bubble expansion due to the density down-ramp and exclude the contributions invoked by beamloading, simulations were performed where macro particles with a forward momentum greater than 10 *m*_*e*_ *c* were removed from the current deposition. This was done in order to get the reference values in Fig. 4.

The datasets generated during and/or analysed during this study are available from the corresponding author on reasonable request.
